# The effect of ultraviolet irradiation compared to oral vitamin D supplementation on blood pressure of nursing home residents with dementia

**DOI:** 10.1186/s12877-021-02538-7

**Published:** 2021-10-19

**Authors:** Bistra I. Veleva, Monique A. A. Caljouw, Astrid Muurman, Jenny T. van der Steen, Victor G. M. Chel, Mattijs E. Numans, Rosalinde K. E. Poortvliet

**Affiliations:** 1 Department of Public Health and Primary Care, Leiden University Medical Center, P.O Box 9600, 2300 RC Leiden, The Netherlands; 2 Woonzorgcentra Haaglanden, Den Haag, The Netherlands

**Keywords:** UVB irradiation, Vitamin D supplementation, Blood pressure, Nursing homes, Dementia

## Abstract

**Background:**

Observational studies have reported an inverse association between ultraviolet (UV) radiation and hypertension. The aim of this study was to assess differences in blood pressure changes between persons with dementia receiving UV light versus vitamin D (VD) supplementation.

**Methods:**

Post-hoc analysis of randomized controlled trial data concerning nursing home residents with dementia (*N* = 61; 41 women, mean age 84.8 years). The participants received half-body UV irradiation, twice weekly over 6 months, at one standard erythema dose (UV group, *n* = 22) or 5600 international units of cholecalciferol once a week (VD group, *n* = 39). Short-term effects were evaluated after 1 month and long-term effects after 3 and 6 months. Differences in blood pressure changes were assessed using linear mixed models.

**Results:**

With the VD group as a reference, the estimated difference in mean change of systolic blood pressure was − 26.0 mmHg [95% confidence interval (CI) -39.9, − 12.1, *p* = .000] at 1 month, 4.5 mmHg (95% CI -6.8, 15.9, *p* = 0.432) at 3 months, and 0.1 (95% CI -14.1, 14.3, *p* = 0.83) at 6 months. The estimated difference in diastolic blood pressure was − 10.0 mmHg (95% CI -19.2, − 0.7, *p* = 0.035) at 1 month, 3.6 mmHg (95% CI -4.1, 11.2, *p* = 0.358) at 3 months, and 2.7 (95% CI -6.8, 12.1, *p* = 0.580) at 6 months.

**Conclusions:**

UV light had only a short-term effect but not a long-term effect on blood pressure reduction compared to VD use in this sample of normotensive to mild hypertensive nursing home residents with dementia. Future studies will be needed to determine the effect of UV light in different samples of the population and especially in a population with hypertension.

**Supplementary Information:**

The online version contains supplementary material available at 10.1186/s12877-021-02538-7.

## Background

Hypertension is a major risk factor for cardiovascular disease (CVD) [1]. Its prevalence increases with older age, reaching 80% in people above the age of 75 [2]. Older people with CVD usually have multiple chronic conditions which are often addressed by guidelines that focus on a single disease, an approach that can increase the risk of inappropriate polypharmacy [3]. In order to reduce the medication burden it may be worthwhile examining readily modifiable risk factors such as insufficient sun exposure and vitamin D (VD) deficiency, both of which play a role in blood pressure homeostasis.

Epidemiological studies have shown that blood pressure correlates with geographical latitude [4], and that sunlight exposure might reduce both blood pressure and CVD [5, 6]. Possible modulators of this effect include VD [7–10], temperature [11], ultraviolet A (UVA) radiation [12, 13] and ultraviolet B (UVB) light radiation [14]. VD (which production in the skin is triggered by UVB) corrects abnormalities in calcium homeostasis and regulates the renin-angiotensin system, both of which play a role in the development of hypertension [15, 16]. It has been proposed that UV light, in addition to its role in the production of VD in the skin, may have a blood pressure regulatory effect that is independent of VD: UVA mediates mobilisation of cutaneous nitric oxide stores to the systemic circulation which works as an endothelial relaxant factor and causes vascular relaxation and vasodilatation [12, 13].

Observational studies suggest an inverse association between sun or UV exposure and blood pressure, an effect that remains even after correcting for temperature, demographic and lifestyle variables and serum 25(OH)D3 concentration [17–19]. There is also some evidence from intervention studies suggesting that UV light might reduce arterial blood pressure but these results are inconsistent [12, 13, 20–24], possibly due to inclusion of different target populations (people with or without hypertension, patients on haemodialysis or healthy volunteers), the UV light spectrum used and the follow-up time. An early effect of UV light exposure on blood pressure was reported by Oplander et al. and Liu et al. [12, 13]. In these two studies healthy volunteers were exposed to a single dose of whole body UVA (20 J/m^2^) for 15 and 22 min, respectively. In the first study, the authors observed a reduction of both systolic and diastolic blood pressure 15 min after the intervention, while in the second study mean diastolic pressure decreased during the intervention and persisted at a lower level for 30 min after the UVA intervention. A randomised trial reported by Krause et al. included 18 patients, aged 26 to 66 years, who were assigned to receive either full-body UVA or UVB irradiation for 6 weeks [22]. UVA had no effect on blood pressure but UVB caused a reduction in both systolic and diastolic blood pressure. Some of these studies attributed the observed effect to the production of VD via UVB light [21, 22], and others to the effect of UVA light on peripheral arterial resistance.

The most consistent body of evidence supporting the effects of VD supplementation, including effects on CVD, is found for older persons with very low serum 25(OH)D3 levels, a finding that supports recommendations for VD supplementation in this population [25–27]. Supplementation of VD is common in nursing home residents with dementia because this group is especially at risk of sun deprivation. Nursing home residents with dementia spend most of their time indoors, and a study by Cutler and Kane showed that of those who are physically able, only 22% actually go outside daily [28]. Whether VD supplementation can completely replace the effect of sun light exposure in maintaining blood pressure homeostasis in nursing home residents with dementia is still not firmly established. Therefore the objectives of this study are:

1. To compare the effect of UV exposure and VD supplementation on blood pressure over time.
2. To compare the effect of UV exposure and VD supplementation on serum 25(OH)D3 levels over time.

## Methods

### Study population and intervention

We conducted a post-hoc analysis of blood pressure data from participants in a multicentre randomized control trial (RCT) that ran for 6 months. The trail was designed to compare the effects of UV light and VD supplementation in terms of well-being of nursing home residents with dementia.

The study population, RCT design and interventions have been described in detail elsewhere [29]. Briefly, participants were recruited from three nursing homes affiliated with the University Network for the Care sector South Holland (UNC-ZH). The RCT was carried out between October 2016 and April 2017 in two nursing homes, and between October 2017 and April 2018 in a third nursing home. Seventy-nine nursing home residents met the inclusion criteria were randomized to the intervention group (UV light, UV group) or standard VD treatment group (control, VD group), which involved supplementation with 5600 International units (IU) cholecalciferol once a week. The intervention consisted of half body UV irradiation with 1 standard erythema dose (SED) two times a week for 8 min. Lamp light emission consisted of UVB-5.013 Wm^− 2^, ultraviolet A (UVA)-4.650 W m^− 2^, ultraviolet C (UVC)-0.00001Wm^− 2^, with UVB accounting for 54.6% of the spectrum. UV treatment was discontinued when participants clearly objected or showed signs of discomfort on two consecutive sessions. They were then removed from the UV exposure group and started on VD capsules. The protocol for the RCT was approved by the Medical Ethical Committee of Leiden University Medical Center (Registration No P16.010) and the study was registered in the Dutch Trial Register (NL5704).

For the post-hoc analyses, blood pressure data were obtained from the medical records of the nursing home residents participating in the RCT. Blood pressure was routinely measured in the first week of each month in the morning after 5 min of quiet rest using an automatic (Omron I-C10/M6, Omron Healthcare Co. Ltd., Kyoto, Japan) sphygmomanometer as a part of standard care. The routine measurements were taken when the nursing home residents were not sick and had no complaints. Serum levels of 25(OH)D3 measured using an electrochemiluminescence immunoassay (ECLIA, Roche diagnostics, Basel, Switzerland) were obtained from the medical records.

### Outcome measures

The primary outcome measure of the post-hoc analysis was the difference in change of systolic and diastolic blood pressure over time between the intervention and control groups and the within group changes over time. Time points of 1 month, 3 months and 6 months after starting the intervention were chosen at which to monitor short-term and long-term effects.

Because of variability in adherence to the intervention in a study population of subjects with dementia, in this post-hoc analysis we created two test situations: 1) a main analysis: all participants exposed to any UV irradiation [UV(all), intervention group] versus VD1 [control group, people randomized to the VD group plus the participants from the UV group who refused irradiation], and 2) an additional analysis concerning all participants exposed to UV for longer than 3 months [UV (exposure> 3 months) group] versus VD2 [control group, people randomized to the VD group plus the participants from the UV group who were exposed to irradiation for less than 3 months].

Differences in the change of serum level of 25(OH)D3 in the intervention versus the control group was a secondary outcome measure. Changes were measured at 3 and 6 months.

### Measurements at baseline

Information on participant’s sociodemographic characteristics (gender, age and skin type) and dementia severity were obtained at baseline. The skin type of each participant was assessed by a dermatologist using the ordinal Fitzpatrick scale which represents a classification of the skin phototypes, based on six categories according to the amount of melanin pigment in the skin, and validated for estimation of the response of different types of skin to UV light [30]..

Dementia severity was assessed using the Bedford Alzheimer Nursing Severity-Scale (BANS-S) [31] which comprises 7 items, scaled 7–28, with a score of 17 or higher indicating severe dementia [32]. For each participant, we took the blood pressure measurement of the month before the start of the intervention as a baseline measurement. The VD status of the participants was estimated based on 25(OH)D3 serum concentrations in nmol/l before starting the intervention.

### Statistical analysis

Statistical analyses were performed with SPSS 23.0 (IBM Corp. Released 2015, Armonk, N.Y., USA). To test differences in basic characteristics between the intervention and control group, we used Pearson’s chi-square test for categorical variables, the unpaired t-test for continuous normally distributed variables and the linear trend test for ordinal variables. A *p*-value < 0.05 was considered statistically significant. Within group differences were measured by a paired t-test and the mean change was determined between baseline and 1 month, 3 months and 6 months. Analysis of the effects of UV light and VD treatments on blood pressure was conducted using linear mixed models for between group differences. In the linear mixed model analyses, time was treated as a categorical variable. Blood pressure was defined as a dependent variable, independent variables were the study groups (control and intervention) and time. Control variables (covariates) were baseline blood pressure for the main outcome and baseline vitamin D for the secondary outcome and for both main and secondary outcome: all baseline characteristics that were significantly different between the intervention and control group. Visual inspection of residual plots did not reveal any obvious deviations from homoscedasticity or normality. The following effects were estimated for the outcome variable: the main effect of the intervention, the main effect of time (at six time points) and the interaction between group and time. The treatment effects were presented at three time points for the systolic and diastolic blood pressure (after 1 month, 3 and 6 months of treatment) and two time points for 25(OH)D3 (after 3 and 6 months of treatment) respectively, as estimated mean scores with 95% confidence interval (CI) and a *p*-value for the adjusted estimated difference between the mean change score (95% CI), with the VD group as reference.

## Results

### Participants

Of the 79 participants included in the RCT, we had blood pressure measurements of 61 participants (33 randomized in the UV group and 28 randomized in the VD group) and we included those 61 participants in the post-hoc analysis. Due to refusal of UV-treatment, we transferred 10 of the participants of the UV-group to the VD group which resulted in the assignment of 23 participants to the UV(all) group and 38 to the VD1 group for the main analysis. On the baseline characteristics between the UV (all) and VD1 groups only a difference in skin type was found (*p* = 0.03) (Table 1). Mean systolic and diastolic blood pressures were 140.5 mmHg (SD 26.0) and 76.6 mmHg (SD 10.1) in de UV group versus 130.3 mmHg (SD 21.5) and 74.1 mmHg (SD 14.2) in the VD1 group (*p* = 0.11). The use of antihypertensive medication was comparable (45.4% in the UV (all) group vs. 30.8% in the VD1 group, *p* = 0.25).

**Table 1:** Characteristics of the participants at baseline by study group

Variable	UV (all) (*n* = 23)	VD1 (*n* = 38)	*p*-value
Gender %, (n)			
Male	26.1 (6)	36.8 (14)	0.39 ^a^
Female	73.9 (17)	63.2 (24)	
Age in years, mean (SD)	84.8 (6.8)	83.5 (7.0)	0.46 ^b^
Fitzpatrick skin scale %, (n)			
1. Always burns easily, never tans	0	2.6 (1)	0.03 ^c^
2. Always burns easily, tans slightly	56.5 (13)	73.7 (28)	
3. Burns moderately, tans gradually	34.8 (8)	23.7 (9)	
4. Burns minimally, tans moderately	0	0	
5. Rarely burns, tans profusely	8.7 (2)	0	
6. Never burns, tans profusely	0	0	
Dementia severity, mean BANS-S (SD)	16.0 (4.0)	15.6 (5.1)	0.75 ^b^
Baseline blood pressure, mmHg			
Systolic, mean (SD)	140.5 (25.4)	130.0 (21.7)	0.09 ^b^
Diastolic, mean (SD)	76.6 (9.9)	74.1 (14.5)	0.48 ^b^
Using antihypertensive medication %, (n)	43.5 (10)	31.6 (12)	0.35 ^a^
Serum 25(OH)D3 levels, nmol/l, mean (SD)	71.6 (24.9)	77.4 (31.9)	0.22 ^b^

*SD* Standard deviation, *BANS-S* Bedford Alzheimer Nursing Severity-Scale, *25(OH)D3* 25-hydroxyvitamin D3

^a^ Pearson’s Chi-squared test used for gender, medication

^b^ Unpaired T-test for age, BANS-S, blood pressure and 25(OH)D3

^c^ Linear trend test

The 25(OH)D3 serum concentration did not differ between the groups (69.6 mmol/l, SD 24.0 in the UV (all) group vs. 78.3 mmol/l, SD 31.9, *p* = 0.32 in the VD1 group). Of the participants in the UV(all) group, 88.9% were VD sufficient (25(OH)D > 50 nmol/L compared to 79.4% in the VD1 group. We adjusted for skin type in the linear mixed model of the main analysis.

For the additional analysis we transferred 10 more patients to the VD (all) group, because they had UV treatment for 3 months or shorter (6 passed away and 4 refused UV treatment and started on VD capsules), so we finally assigned 13 participants to the UV(> 3 months) group and 48 patients to the VD2 group. The baseline characteristics of the participants in the additional analysis showed no difference with exception of the serum 25(OH)D3 concentration which was significantly lower in the UV(> 3 months) group, *p* = 0.04 (Additional file 1). We adjusted for this in the linear mixed model of the additional analysis.

### Effect of UVB treatment on systolic blood pressure

After 1 month of treatment, the mean systolic blood pressure in the UV(all) group was 24.5 mmHg lower (95% CI 7.6, 41.3, *p* = 0.008) than at baseline (Table 2). By contrast, mean systolic blood pressure in the VD1 group did not change significantly, with a mean change of 6.2 mmHg (95% CI − 10.1, 22.7, *p* = 0.416). The adjusted mean change difference between the two groups, with the VD1 group as a reference, after 1 month of treatment, was − 26.0 mmHg (95% CI − 39.9, − 12.1, *p* = .000) (Table 3). At 3 and 6 months there was neither within group difference nor between group difference in systolic blood pressure of the control and intervention group.

**Table 2:** Within group differences between baseline and 1, 3 and 6 months: Paired T-test

Group	Period	Outcome variable	Mean Change	95% CI of the difference		***p***-value
				Lower	Upper	
**VD1**	1–0 month, *n* = 11	Systolic BP	6,2	-10,1	22.7	,416
	3–0 month, *n* = 30	Systolic BP	-3,8	−11.3	3,6	,300
	6–0 month, *n* = 17	Systolic BP	− 4,9	−18,2	8.4	,444
	1–0 month, n = 11	Diastolic BP	3,8	−7,1	14.7	,455
	3–0 month, n = 30	Diastolic BP	−1,2	−7,5	5.1	,702
	6–0 month, n = 17	Diastolic BP	−4,6	−13,4	4.1	,278
	3–0 month, *n* = 24	25(OH)D3	−4,6	−11,4	2.2	,172
	6–0 month, *n* = 21	25(OH)D3	4,9	−2,9	12,8	,208
**UV(all)**	1–0 month, n = 13	Systolic BP	−24,4	−41,9	−7,6	,008
	3–0 month, *n* = 13	Systolic BP	−7,1	−22,9	8.6	,342
	6–0 month, *n* = 8	Systolic BP	−7,7	−26,7	11,2	,366
	1–0 month, n = 13	Diastolic BP	−7,1	−15,0	0.9	,076
	3–0 month, n = 13	Diastolic BP	0,4	−6,0	6,8	,898
	6–0 month, n = 8	Diastolic BP	2,7	−5.1	10.6	,437
	3–0 month, n = 13	25(OH)D3	−6,3	−15,4	2.9	,163
	6–0 month, *n* = 9	25(OH)D3	−11,5	−23.0	−0,02	,050

*95% CI* 95% Confidence interval, *Systolic BP* Systolic blood pressure, *Diastolic BP* Diastolic blood pressure, *25(OH)D3* serum 25-hydroxyvitamin D3

UV (all): the group of the people, received any UVB radiation, VD1 group: people randomized in VD group plus the participants from the UV group who have refused irradiation

**Table 3:** Estimated marginal group means and *p*-values, based on mixed model analysis^∗^

	1 month			3 months			6 months			***p***-value	Pt	Pgt
	Estimated mean	Adjusted MD	***p***-value	Estimated mean	Adjusted MD	***p***-value	Estimated mean	Adjusted MD	***p***-value	Pg		
	score (95%CI)	(95%CI)		score (95%CI)	(95%CI)		score (95%CI)	(95%CI)				
**Systolic BP**												
**Main analysis**										0.612	0.811	0.002
UV (all), n = 23	117.4 (107.9, 126.8)	−26.0 (−39.9, −12.1)	.000	132.1 (122.8, 141.5)	4.5 (−6.8, 15.9)	0.432	128.3 (116.6, 139.9)	0.1 (−14.1, 14.3)	0.832			
VD1, n = 38	143.4 (133.6, 153.2)			127.6 (121.4, 133,6)			128.2 (120.2, 136.2)					
**Additional analysis**										0.256	0.642	0.064
UV (> 3 months), n = 13	116.8 (103.5, 130.1)	−22.3 (−38.7, −5.9)	0.008	127.6 (117.0, 138.3)	−3.1 (−15.7, 9.6)	0.632	123.3 (109.2, 137.4)	−7.0 (−23.5, 9.4)	0.400			
VD2, n = 48	139.1 (129.5, 148.7)			130.7 (124.1, 137.4)			130.3 (121.9, 138.7)					
**Diastolic BP**												
**Main analysis**										0.692	0.738	0.106
UV (all), n = 23	69.4 (63.0, 75.7)	−10.0 (−19.2, −0.7)	0.035	77.3 (71.2, 83.7)	3.6 (−4.1, 11.2)	0.358	76.4 (68.7, 84.2)	2.7 (−6.8, 12.1)	0.580			
VD1, n = 38	79.3 (72.8, 85.9)			73.8 (69.6, 78.0)			73.8 (68.4, 79.1)					
**Additional analysis**										0.580	0.802	0.712
UV (> 3 months), n = 13	70.0 (61.3, 78.7)	−6.6 (−17.4, 4.2)	0.227	75.7 (68.6, 82.8)	0.9 (−7.5, 9.3)	0.834	74.6. (65.3, 83.9)	0.9 (−10.0, 11.7)	0.878			
VD2, n = 48	76.6 (70.3, 82.9)			74.8 (70.3, 79.1)			73.7 (68.2, 79.3)					
**25(OH)D3**												
**Main analysis**										0.037	0.230	0.006
UV (all), n = 23				67.3 (58.3, 76.3)	- 5.9 (−17.3, 5.4)	0.299	64.0 (54.3, 73.5)	- 17.5 (−29.3, −5.7)	0.004			
VD1,n = 38				73.2 (66.8, 79.6)			81.4 (74.8, 87.9)					
**Additional analysis**										0.076	0.395	0.04
UV (> 3 months), n = 13				67.5 (57.7, 77.2)	−5.3 (−16.9, 6.4)	0.368	64.6 (53.9, 75.3)	−15.0 (−27.4., −2.5)	0.019			
VD2, n = 48				72.7 (66.6, 78.9)			79.6 (73.3, 85.8)					

*Systolic BP* Systolic blood pressure, *Diastolic BP* Diastolic blood pressure, *25(OH)D3* serum 25-hydroxyvitamin D3

*The mixed model analysis adjusted for the baseline of the outcome measures shows the *p*-values for the intervention (UVB) versus control (VD) condition UV (all): the group of the people, received any UVB radiation, VD1 group: people randomized in VD group plus the participants from the UV group who have refused irradiation UV (> 3 months): all participants having had UV exposure longer than 3 months, VD2 group: people randomized in the VD group plus all the participants from the UV group having had irradiation shorter than 3 months. Main analysis: VD1 - control group and UV (all) -intervention group, adjusted for the baseline of the outcome measures and skin type. Additional analysis: VD2 – control group and UV (> 3 months)) - intervention group, adjusted for the baseline of the outcome measures (Pg), the overall time effect (Pt) and the interaction effect of group and time (Pgt). The treatment effect is presented as adjusted mean difference (MD) between the VD and UV group for each time point with VD group as reference category

### Effect of UVB treatment on diastolic blood pressure

After 1 month of treatment, the mean diastolic blood pressure in the UV(all) group was 7.1 mmHg (95% CI -15.0, 0.9, *p* = 0.076) lower than baseline versus 3.8 mmHg (95% CI -7.1, 14.7, *p* = 0.455) higher than baseline in the VD1 group, but neither change was statistically significant. The adjusted mean change difference between the two groups, with the VD1 group as a reference, was − 10.0 mmHg (95% CI -19.2, − 0.7, *p* = 0.035). At 3 and 6 months, there was no statistically significant within and between group differences in diastolic blood pressure.

### Additional analysis

In an additional analysis restricted to participants who were exposed to UV for longer than 3 months the results were similar [UV (exposure > 3 months), *n* = 13, VD2, *n* = 48] (Table 3). The adjusted difference in the change in systolic blood pressure between the groups, with the VD2 group as reference, was − 22.3 mmHg (95% CI -38.7, − 5.9, *p* = 0.008) after 1 month, − 3.1 mmHg (95% CI -15.7, 9.6, *p* = 0.632) at 3 months and − 7.0 (95% CI -23.5, 9.4, *p* = 0.400) at 6 months. The adjusted difference in the change in diastolic blood pressure between the groups, with the VD2 group as reference, was not significant at all time points.

### Secondary outcomes

At 3 months, there were no within or between group differences in serum concentrations of 25(OH)D3 in the intervention or control group in either the main or additional analysis (Tables 2 and 3). At 6 months, however, the serum concentration of 25(OH)D3 in both UV groups [UV(all) estimated mean 64.0 nmol/l (95% CI 54.3, 73.5) and UV (> 3 months) estimated mean 64.6 (95% CI 57.7, 77.2)] was lower than in the VD groups [VD1 estimated mean 81.4 nmol/l (95% CI 74.8, 87.9) and VD2 estimated mean 79.6 nmol/l (95% CI 73.3, 85.8)]. The estimated difference between the mean scores was − 17.5 nmol/l (95% CI -29.3, − 5.7, *p* = 0.004) and − 15.0 nmol/l (95% CI -27.4, − 2.5, *p* = 0.019), respectively. The overall group effect estimating for the change in the difference between the two groups over the whole period was significant in the main analysis (*p* = 0.037) but not significant in the additional analysis (*p* = 0.076).

## Discussion

This post hoc analysis found no sustained effect of UV light compared to VD supplementation on blood pressure in nursing home residents with dementia aged 70 years and older. A reduction of blood pressure was seen in the UV group in the first month of treatment but was no longer observed at three and six months.

There are two frequently mentioned hypotheses regarding how UV light might influence blood pressure: the Vitamin D (VD) hypothesis and Nitric Oxide (NO) hypothesis. The VD hypothesis assumes that UVB light triggers the production of VD, which then exerts antihypertensive and vasculoprotective effects [33]. Possibly this is an indirect mechanism which is a part of a complex process in maintaining blood pressure homeostasis. In our study the baseline levels of the serum 25(OH)D3 in the intervention and control group were comparable. After 3 months there was also not a significant change in serum 25(OH)D3 concentration in either groups. The reduction of blood pressure in the first month of the intervention in the UV(all) group cannot be explained with the VD-hypothesis. The NO hypothesis assumes that UVA mobilizes cutaneous NO stores [12] or NO from intracutaneous photolabile nitric oxide derivatives [13] to the systemic circulation, resulting in a rapid and direct effect of endothelial relaxation and subsequent vascular relaxation and vasodilatation. Mobilisation of NO stores from the skin to the circulation when irradiated by UV light might explain the reduction of blood pressure in the UV group during the first month of our study. However, the fact that this effect was not sustained in the following months of our study might be explained by the hypotheses underlying the mechanisms of development of tolerance to nitrates: the “metabolic” theory which suggests decreased activity of the NO released in the NO-induced vasodilatation (end-organ tolerance) and the “functional” theory highlighting the counterregulatory mechanisms marked by neurohumoral activation, increased catecholamine release, sodium retention and intravascular volume expansion [34, 35].. Moreover, the old and frail condition of the nursing home residents in our study may have also influenced the depletion-repletion kinetics of the cutaneous NO pool. Our study population was also normotensive to mildly hypertensive (according to the definition of the European Society of Cardiology [36]), with 45.4% of the participants in the UV group and 30.8% in the VD group using antihypertensive medication, which can trigger cardiovascular and central regulatory mechanisms that further limit blood pressure reduction.

We only hypothesize but we do not know why the effect of UV light on blood pressure reduction in our study was of a short duration. People with hypertension have frequently endothelial dysfunction and decreased NO synthesized from the vascular endothelium [37]. Using the cutaneous release of NO in controlling blood pressure is an attractive option. NO is a multipotent molecule which stimulates a cascade of reactions which result in vasodilatation of vascular smooth muscle cells, prevention of platelet adhesion and aggregation and a range of anti-inflammatory and anti- proliferative reactions preventing atherosclerosis [38]. Having in mind the above mentioned mechanisms which might have determined the short duration of UV effect on blood pressure, it is interesting to replicate the study in a group of younger (better depletion-repletion kinetics) and hypertensive patient’s not using medication (to possibly avoid the counterregulation). For old people with dementia using antihypertensive medication and going outside more frequently, it might be relevant to check blood pressure in the summer months and eventually consider to stop or reduce the medication. Although patients with dementia have no increased vulnerability to blood pressure lowering treatment [39] and a good control of blood pressure may prevent disability from stroke [40, 41], maintaining the 150–130 mmHg on-treatment systolic blood pressure values are the safety range for optimal physical and cognitive functioning [42–44].

A major strength of this post hoc analysis was the use of repeated measurements for the outcome variables of participants. We had a control group and the participants were randomized at random initially. The randomisation that we used in the test situations created in the post-hoc analysis was not based on selection on the outcome variables. We used mixed linear model analysis which provides the flexibility of modelling not only the means of the data but their variances and covariances as well. We have also corrected for the baseline measurements. With the linear mixed modelling we looked at the difference in the changes between the control and intervention group, but we used also the parametric test for controlling for the within group changes.

This post-hoc analysis has some limitations. We used data of our RCT for a secondary data analysis. Blood pressure measurements were taken from patients’ files and not measured according to a standardized protocol, a single measurement was performed per time point. We had also missing data which was partially mitigated carrying out a linear mixed model analysis, corrected for baseline blood pressure. The number of the participants was small (wide confidence intervals for the findings) and the study may have had limited power to detect a clinically important difference between the intervention and control group. We had no data on the natural UV exposure time and dietary vitamin D.

## Conclusion

This post hoc analysis found a short-term effect (at 1 month) but not a long term effect (at 3 and 6 months) of UV regarding systolic and diastolic blood pressure reduction in a VD-sufficient population of nursing home residents with dementia. Future larger studies with an RCT design should investigate the effect of UV in both the short and long-term and also in different populations (VD-sufficient vs. VD-insufficient, hypertensive vs. normotensive). This will contribute to understand better the association between ultraviolet light and hypertension and the role of sun exposure as a modulator in CVD risk management which is of crucial importance for the population of frail older people who are particularly deprived of sun exposure.

## Supplementary Information

**Additional file 1: Table A1** Characteristics of the participants at baseline by study group (additional analysis).

## Data Availability

The datasets used and/or analysed during the current study are available from the corresponding author on reasonable request.

## Abbreviations

CVD: Cardiovascular disease
VD: Vitamin D
UV: Ultraviolet
UVA: Ultraviolet A
UVB: Ultraviolet B
UVC: Ultraviolet C
25(OH)D3: 25-hydroxyvitamin D3
NO: Nitric oxide
RCT: Randomized control trial
UNC-ZH: University Network for the Care sector South Holland
IU: International units
SED: Standard erythema dose
BANS-S: Bedford Alzheimer Nursing Severity-Scale
SD: Standard deviation
