# Supplementary material for: The effect of ultraviolet irradiation compared to oral vitamin D supplementation on blood pressure of nursing home residents with dementia
Source: BMC Geriatr. 2021 Oct 19;21:577. doi: 10.1186/s12877-021-02538-7 (PMC8524945; doi:10.1186/s12877-021-02538-7)
Supplement: Supplementary file 1 — Additional file 1: Table A1 Characteristics of the participants at baseline by study group (additional analysis). [file 12877_2021_2538_MOESM1_ESM.docx]

**Table A1 Characteristics of the participants at baseline by study group (additional analysis)**

| **Variable** | **UV (>3 months)**  **(n=13)** | **VD2**  **(n=48)** | **p-value** |
| --- | --- | --- | --- |
| **Gender %, (n)**  **male**  **female** | 15.4 (2)  84.6 (11) | 37.5 (18)  62.5 (30) | 0.13 ^a^ |
| **Age in years, mean (SD)** | 84.6 (6.5) | 83.8 (7.1) | 0.75 ^b^ |
| **Fitzpatrick skin scale %,(n)**  **1.always burns easily, never tans**  **2.always burns easily, tans slightly**  **3.burns moderately, tans gradually**  **4.burns minimally, tans moderately**  **5.rarely burns, tans profusely**  **6.never burns, tans profusely** | 0  53.8 (7)  38.5 (3)  0  7.7 (1)  0 | 2.1 (1)  70.8 (34)  25 (12)  0  2.1 (1)  0 | 0.13 ^c^ |
| **Dementia severity, mean BANS-S (SD)** | 14.9 (4.5) | 15.9 (4.8) | 0.50 ^b^ |
| **Baseline blood pressure, mmHg**  **Systolic, mean (SD)**  **Diastolic, mean (SD)** | 143.4 (26.1)  75.5 (10.5) | 131.4 (22.4)  74.9 (13.5) | 0.32 ^b^  0.56 ^b^ |
| **Using antihypertensive medication %,(n)** | 46.2 (6) | 33.3 (16) | 0.39 ^a^ |
| **Serum 25(OH)d3 levels, nmol/l, mean (SD)** | 65.2 (17.4) | 78.3 (31.8) | 0.04 ^b^ |

SD, Standard deviation, BANS-S, Bedford Alzheimer Nursing Severity-Scale, 25(OH)D3, 25-hydroxyvitamin D3

a - Pearson’s Chi-squared test used for gender, medication

b - Unpaired T-test for age, BANS-S, blood pressure and 25(OH)D3

c –Linear trend test
